# Long noncoding RNA expression profile from cryptococcal meningitis patients identifies DPY19L1p1 as a new disease marker

**DOI:** 10.1111/cns.13109

**Published:** 2019-02-14

**Authors:** Lei Zhang, Wen‐Jie Fang, Ke‐Ming Zhang, Wei‐Wei Jiang, Min Chen, Wan‐Qing Liao, Wei‐Hua Pan

**Affiliations:** ^1^ Department of Dermatology and Venereology, Changzheng Hospital Second Military Medical University Shanghai China; ^2^ Shanghai Key Laboratory of Molecular Medical Mycology, Shanghai Institute of Medical Mycology, Changzheng Hospital Second Military Medical University Shanghai China

**Keywords:** biomarker, cryptococcal meningitis, DPY19L1p1, long noncoding RNAs

## Abstract

**Aims:**

LncRNAs play a vital role in the pathological and physiological process. This study aimed to explore the involvement of lncRNAs in cryptococcal meningitis.

**Methods:**

Microarray was performed in cryptococcal meningitis patients, and then, GO and KEGG pathways were analyzed. Coexpression relationship between lncRNA and mRNA was explored. The expressions of the lncRNAs and mRNAs, and their changes after treatment were detected by PCR.

**Results:**

A total of 325 mRNAs (201 upregulated and 124 downregulated) and 497 lncRNAs (263 upregulated and 234 downregulated) were identified. The top three enriched GO terms for the mRNAs were arachidonic acid binding, activin receptor binding, and replication fork protection complex. The top three pathways in KEGG were asthma, one carbon pool by folate, and allograft rejection. A total of 305 coexpression relationships were found between 108 lncRNAs and 87 mRNAs. LncRNA‐DPY19L1p1 was significantly increased in patients and decreased after treatment. ROC analysis revealed DPY19L1p1 was a potential diagnostic marker (AUC_ROC_ = 0.9389). Furthermore, the target genes of DPY19L1p1 in *cis* or *trans* regulation were mainly involved in immune‐related pathways like the interleukin signaling pathway.

**Conclusions:**

This study analyzed the differential lncRNA profile in cryptococcal meningitis patients and revealed DPY19L1p1 could be used for treatment evaluation and disease diagnosis.

## 1 INTRODUCTION

Cryptococcal meningitis, a severe central nervous system infection diseases, accounts for 15% of the AIDS‐related mortality worldwide.^1^ In immunocompetent individuals, *Cryptococcus neoformans* primarily causes asymptomatic clinical manifestations or latent infection; most people have a history of *C. neoformans* exposure in early childhood.^2^ With the growing population of patients with AIDS or organ transplantation history, the incidence of cryptococcosis has tended to increase.^3^ Because *Cryptoccocci *are opportunistic fungi, the progress and prognosis of cryptococcosis predominantly depend on the interplay between the host immune response and the fungus*. *However, most current studies used murine models or healthy human cells to assess the aberrant levels of immune‐related factors. Although a recent study has shown the differentially expressed genes of *Cryptoccoccus* at the site of human meningitis infection,^4^ the key immune system regulators in patients with cryptococcal meningitis are poorly known.

LncRNAs are a large family of noncoding RNAs, accounting for approximately 85% of the transcribed human genome;^5^ LncRNAs are widely expressed in a variety of immune cells, including T cells, B cells, monocytes, and dendritic cells and can function as key regulators of immunogene transcription.^6^ The precise regulation of lncRNAs is important in maintaining homeostasis. Abnormally expressed lncRNAs participate in many immune‐related diseases, such as autoimmune diseases,^7^ bacterial diseases, and viral diseases.^8^, ^9^ The fungal lncRNA RZE1 has been reported to control the *Cryptococcus *yeast‐to‐hypha transition by regulating the key morphogenetic regulator Znf2,^11^ indicating that lncRNAs are involved in fungal virulence. However, little is known about the role of host lncRNAs during fungal infection, especially during *Cryptococcus *infection, in clinical settings.

In this study, based on microarray and bioinformatic analysis, for the first time, we reported the differential lncRNA profile in cryptococcal meningitis patients and revealed DPY19L1p1 could be used not only in treatment evaluation but also for disease diagnosis through receiver operating characteristic curve analysis.

## 2 METHODS

### 2.1 Subjects

A 5 mL volume of venous blood was collected from twenty cryptococcal meningitis patients and eighteen healthy donors from the Changhai hospital and Changzheng hospital. The diagnosis of cryptococcal meningitis was based on India ink staining and/or positive culture of *C. neoformans *from cerebrospinal fluid.^12^ The age and gender of the healthy control and the cryptococcal meningitis groups were not significantly different. All subjects were confirmed to be HIV‐negative. Ficoll density gradient centrifugation was used to harvest peripheral blood monocytes (PBMCs) as previously described.^13^ PBMCs were then stored in liquid nitrogen. Informed consent was obtained from all subjects, and this study was approved by the ethics committees of the Changhai hospital and Changzheng hospital (Shanghai, China).

### 2.2 RNA extraction and chip analysis

Total RNA was extracted and purified using a miRNeasy Mini Kit following the manufacturer's instructions, and RNA integrity was evaluated by the RNA integrity number (RIN) with an Agilent Bioanalyzer 2100. For the chip analysis, total RNA was amplified and labeled by a Low Input Quick Amp WT Labeling Kit following the manufacturer's instructions. Labeled cRNA was purified by an RNeasy Mini Kit. Each slide was hybridized with 1.65 μg Cy3‐labeled cRNA using a Gene Expression Hybridization Kit in a hybridization oven to the manufacturer's instructions. After hybridization for 17 hours, the slides were washed in staining dishes with a Gene Expression Wash Buffer Kit following the manufacturer's instructions. The slides were scanned by an Agilent scanner the default settings (dye channel: green, scan resolution = 3 μm, PMT 100%, 20 bit). The data were extracted with Feature Extraction software 10.7. The raw data were normalized by the quantile algorithm of the limma package in R.

### 2.3 Gene ontology and Kyoto encyclopedia of genes and genomes analyses

GO analysis covers three domains as follows: cellular component, molecular function, and biological process. The GO and KEGG enrichment analyses were performed with Fisher's exact test based on the data package ClusterProfiler (R/bioconductor); the selection criterion was that the fold change in the gene expression must be ≥2 with a *P*‐value of <0.05. The enrichment factor (enrich_factor) was defined as follows: enrich_ factor = (number of differentially expressed genes in the GO term/total number of differentially expressed genes)/(total number of genes in the database term/total number of genes in the database).

### 2.4 Correlation analysis between lncRNAs and mRNAs

The network between lncRNAs and mRNAs was constructed based on the correlation analysis of differentially expressed lncRNAs and protein‐coding genes. For each lncRNA‐mRNA pair, Pearson correlation was performed to assess the correlation. Pairs for which the absolute value of the Pearson correlation coefficient was not <0.80 and the *P*‐value was <0.05 were selected to generate the network using Cytoscape (National Resource for Network Biology).

### 2.5 Real‐time PCR

For real‐time PCR, total RNA was extracted using TRIzol reagent, and qRT‐PCR was performed to verify the RNA sequencing (RNA‐seq) data using SYBR Green (TaKaRa, Japan) and an ABI 7500 SDS system (Applied Biosystems, USA). The primer sequences are shown in Table 1. Beta‐actin was used as the endogenous control. The relative expression value of the gene of interest was calculated via the 2^–ΔΔCt^ method.

**Table 1:** Primers used for real‐time PCR

Primer	Forward (5′‐3′)	Reverse (5′‐3′)
CAMP	GGCTGGTGAAGCGGTGTAT	TGGGTACAAGATTCCGCAAAAA
CRISP3	CCTGTTCCACCGGTTTTGTTTT	TTGCACTTGTGTTTGGGTGG
LTF	CCCAGGAACCGTACTTCAGC	GTGCCACAACGGCATGAGA
OLR1	TTGGATGCCAAGTTGCTGAAAA	ATGGGTAGCTGGGGTTCCT
BPI	GAAGGCATGTCCATTTCGGCT	TCGAAGCGCAGACTCAATTTT
CTSG	GAGTCAGACGGAATCGAAACG	CGGAGTGTATCTGTTCCCCTC
PGLYRP1	GCCTGCCCTTACGCTATGTG	CAGGAAGTTGTAGCCCACGTC
ARG1	TGGACAGACTAGGAATTGGCA	CCAGTCCGTCAACATCAAAACT
OLFM4	ACTGTCCGAATTGACATCATGG	TTCTGAGCTTCCACCAAAACTC
CEACAM8	TCTATCGTGTCAACCCCAAAT	AGATGCTGTTACTGTCAGCCA
ECRP	TTCTCATAGGAGCCACAGCG	TACTGATGGACGTCAAACCCC
LINC00968	GTCCACCCACTGGTCCATTT	GTGCTGAGCTGTCTGGAAGT
DPY19L1p1	TGGGAAGCACCGCTTTACAT	CTAGGAGCTCTGTGAGGGGT
DEFA8P	GCACCTGCAGATGAGATTCCT	CTGAAGCAGTATGGGTAGCGT
DEFT1P2	GACTCAGCGAGAGGCTTGAG	AGCAGATCCGGTGGAGTGTA
DDX11L10	CTTCCCCAGCATCAGGTCTC	TCAGATTCAGGCCAACAGCC
MTMR9LP	GTCTTGAGCACTTGCTCCCT	ACATGTTACAGTCAGCGGCA
β‐ACTIN	AGCGAGCATCCCCCAAAGTT	GGGCACGAAGGCTCATCATT

### 2.6 Statistical analysis

Differential comparisons between groups were made by a t test. A *P*‐value of <0.05 was considered statistically significant. All statistical analysis was performed with GraphPad Prism software (La Jolla, CA, USA).

## 3 RESULTS

### 3.1 Clinical characteristics of cryptococcal meningitis patients

Twenty cryptococcal meningitis patients (eight female, twelve male; age range: 21‐56 years, median age: 43 years) and eighteen healthy controls (six female, twelve male; age range: 19‐50 years, median age: 38 years) were included in this study. The clinical information is shown in Table 2. PBMCs from three randomly selected cryptococcal meningitis patients (P1‐P3) and three healthy controls were used for the microarray analysis.

**Table 2:** Clinical parameters of the cryptococcal meningitis patients

No.	Sex	Age	BMI	CD4%	Nutritional status	Previous history of diseases	Previous history of immunosuppressive agent use	Titer in latex agglutination test (blood)
P1	F	41	22.1	42	Good	None	None	1:40
P2	F	21	19.3	32	Medium	None	None	1:1280
P3	M	56	22.0	37	Medium	None	None	1:40
P4	M	42	21.2	47	Medium	Hospital‐acquired pneumonia	Dexamethasone	1:160
P5	M	33	24.0	37	Good	None	None	1:160
P6	M	35	19.6	39	Medium	None	None	1:640
P7	F	56	20.2	NA	Medium	Liver cancer	None	1:40
P8	F	31	19.2	32	Medium	Hepatitis B	None	1:40
P9	M	21	21.3	NA	Medium	Tuberculosis	None	1:640
P10	M	47	23.7	39	Medium	Type 2 diabetes	None	1:1280
P11	M	51	20.1	35	Poor	None	None	1:2560
P12	M	53	23.4	36	Good	None	None	1:640
P13	F	47	17.7	NA	Medium	None	None	1:40
P14	F	37	19.7	33	Medium	Systemic lupus erythematosus	Methylprednisolone, dexamethasone	1:40
P15	M	44	19.3	49	Medium	None	None	1:160
P16	F	54	20.5	32	Medium	Acute rapidly progressive glomerulonephritis	Prednisone, methylprednisolone	1:40
P17	F	34	23.1	NA	Good	None	None	1:1280
P18	M	50	24.0	33	Good	None	None	1:640
P19	M	27	23.4	NA	Good	None	None	1:640
P20	M	49	22.5	NA	Good	None	None	1:1280

BMI, body mass index; M, male; NA, not available.

### 3.2 Differential expression profiles of lncRNAs and mRNAs between cryptococcal meningitis patients and healthy controls

**Figure 1:**
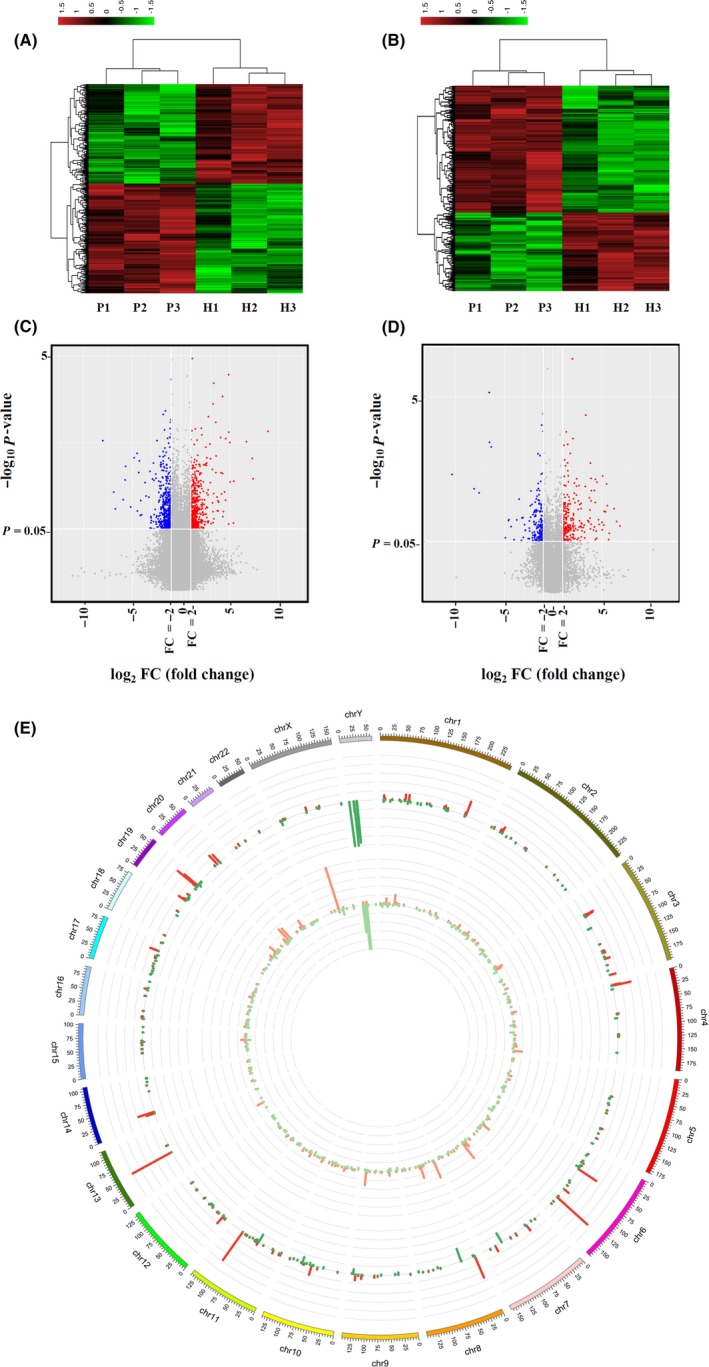
Differential expression of lncRNAs and mRNAs between cryptococcal meningitis patients (n = 3) and healthy controls (n = 3). A, B, Heat map of lncRNA and mRNA expression; C, D, volcano plot of lncRNA and mRNA expression; the red dots represent upregulated RNAs with a *P*‐value <0.05 and a fold change ≥2, and the blue dots represent downregulated RNAs with a *P*‐value <0.05 and a fold change ≤0.5; E, Chromosomal distributions of differentially expressed lncRNAs and mRNAs chromosomes, differential mRNA expression, and differential lncRNA expression are presented from the outside circle to the inside circle. Green represents downregulation, red represents upregulation, and the height of the bars represents gene enrichment. CN, cryptococcal meningitis; HC, healthy controls

In this study, 18 853 mRNAs (8951 upregulated and 9902 downregulated) were differentially expressed, and 68 423 lncRNAs (33 999 upregulated and 34424 downregulated) were differentially expressed (datasets are available on request). We used a fold change of ≥2 and a *P‐*value of <0.05 as the cutoff to determine the profile of significantly differentially expressed genes. A total of 325 mRNAs (201 upregulated and 124 downregulated) (Figure 1A,C) and 497 lncRNAs (263 upregulated and 234 downregulated) (Figure 1B,D) were identified. In addition, circos plots were generated to demonstrate the chromosomal distribution of these differentially expressed lncRNAs and mRNAs (Figure 1E).

### 3.3 GO and KEGG pathway analyses of mRNAs

**Figure 2:**
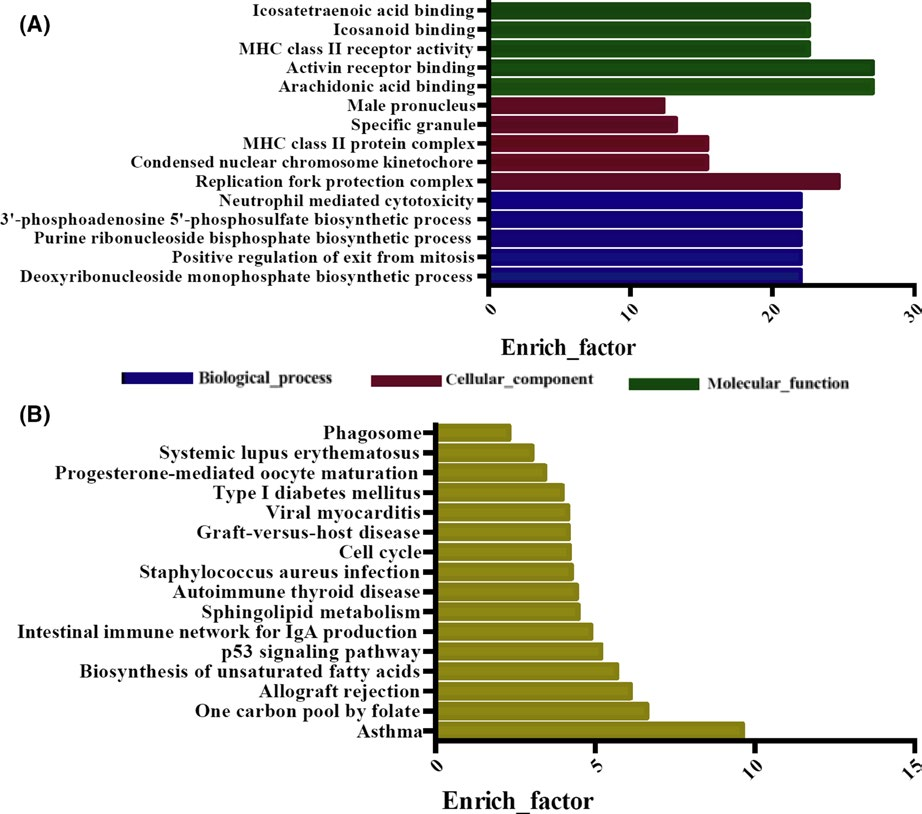
GO and KEGG analyses of the differential mRNA expression profile. A, GO; B, KEGG

GO (Figure 2A) and KEGG pathway (Figure 2B) analyses were used to identify the functional implications of these differentially expressed mRNA. The top five GO terms were as follows: arachidonic acid binding (GO:0050544), activin receptor binding (GO:0070697), replication fork protection complex (GO:0031298), MHC class II receptor activity (GO:0032395), and icosanoid binding (GO:0050542). The top five KEGG pathways were as follows: asthma (hsa05310), one carbon pool by folate (hsa00670), allograft rejection (hsa05330), biosynthesis of unsaturated fatty acids (hsa01040), and p53 signaling pathway (hsa04115).

### 3.4 Classification of differentially expressed lncRNAs

**Figure 3:**
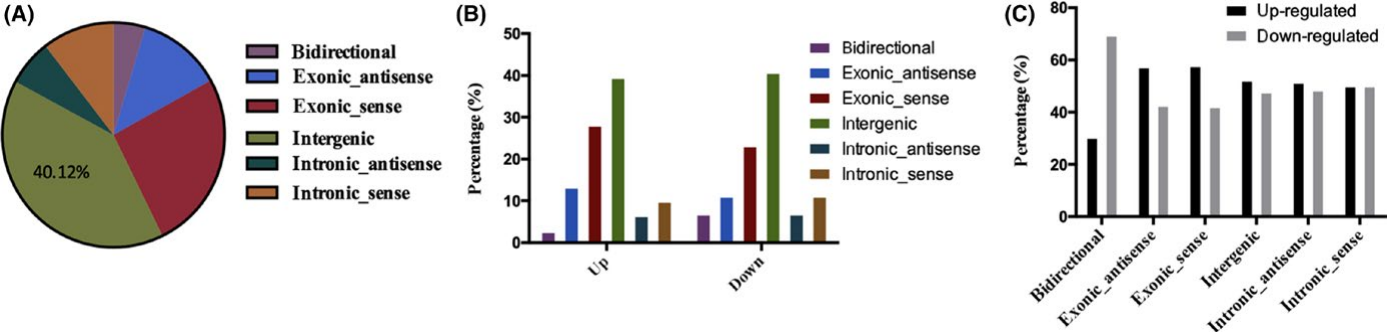
Classification and percentages of differentially expressed lncRNAs. A, Proportions of differentially expressed lncRNAs within each classification; B, proportions of the different types of lncRNA among upregulated and downregulated lncRNAs; C, proportions of upregulated and downregulated lncRNAs of each type

The type of lncRNA can indicate its regulatory function. As shown in Figure 3A, the majority of the lncRNAs were intergenic (40.12%), followed by exonic sense (25.81%), exonic antisense (12.3%), intronic sense (10.48%), intronic antisense (6.65%), and bidirectional (4.64%). In both the upregulated and downregulated subsets of lncRNAs (Figure 3B), intergenic lncRNAs were the most prevalent (39.54%, 40.77%), followed by exonic sense (28.14%, 23.18%), exonic antisense (13.31%, 11.16%), intronic sense (9.89%, 11.16%), intronic antisense (6.46%, 6.87%), and bidirectional (2.66%, 6.87%) lncRNAs. The numbers of upregulated and downregulated lncRNAs (Figure 3C) were quite similar for each type of differentially expressed lncRNA except bidirectional lncRNAs, for which the number of downregulated genes was more than twice the number of upregulated genes.

### 3.5 LncRNA and mRNA coexpression analysis

**Figure 4:**
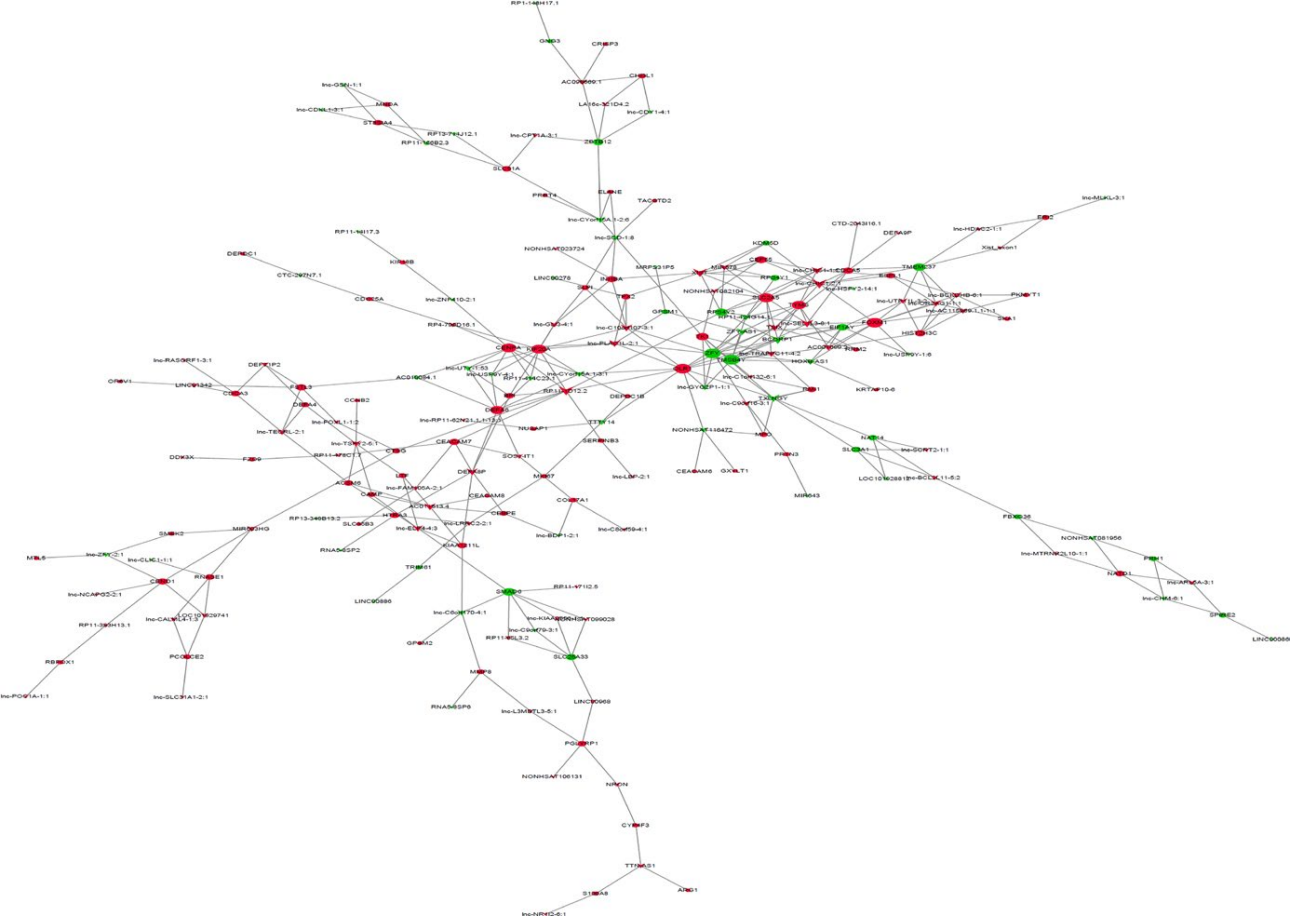
Coexpression network of mRNAs and lncRNAs. The triangles represent lncRNAs, and the circles represent mRNAs; red indicates upregulation, and green indicates downregulation. The dots represent mRNAs, and the arrows represent lncRNAs

Coexpression network analysis is another method used to predict lncRNA function. In this study, 305 coexpression relationships were found between 108 lncRNAs and 87 mRNAs (Figure 4). Many mRNAs, including those coding for genes such as smad family member 6 (SMAD6), centromere protein A (CENPA), kinesin family member 20A (KIF20A), defensin alpha 6 (DEFA6), and oxidized LDL receptor 1 (OLR1), were found to interact with several lncRNAs. In addition, connections were also found between several lncRNAs, such as RP11‐11D12.2, and several mRNAs.

### 3.6 Validation of differential expression of mRNA and lncRNA and dynamic changes after treatment

**Figure 5:**
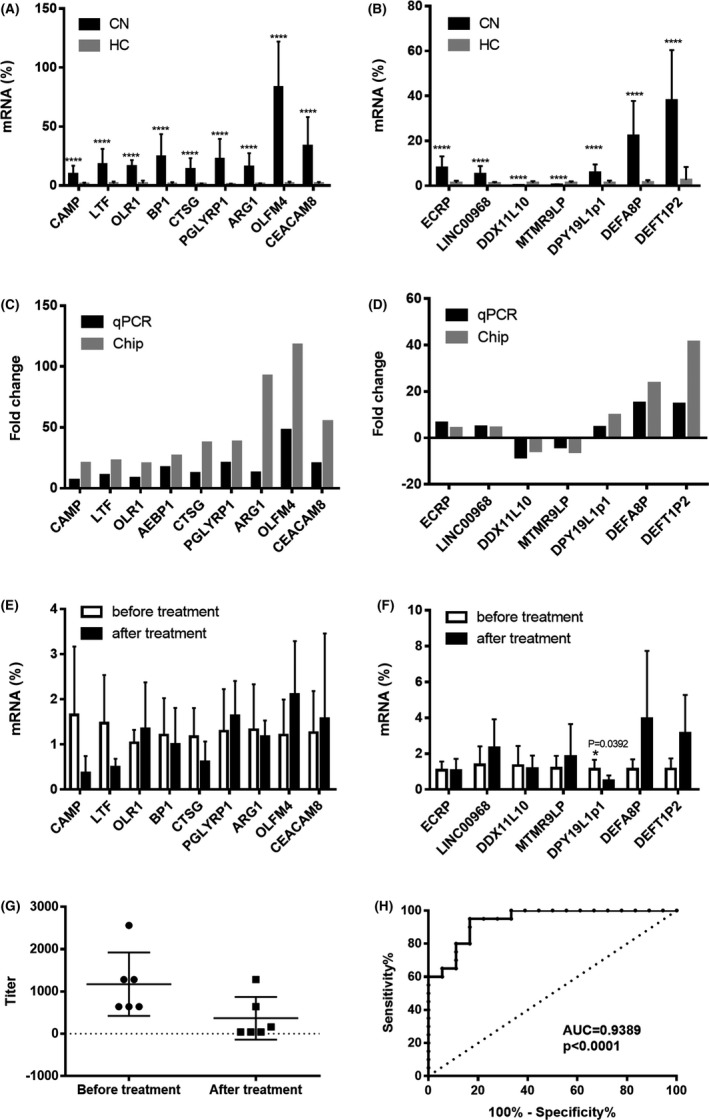
Validation microarray results and evaluation the clinical value of lncRNA DPY19L1p1 by real‐time PCR. A, B. Relative expression of mRNAs (A) and lncRNAs (B); C, D, comparison of fold changes in mRNA (C) and lncRNA (D) expression between real‐time PCR and microarray; E, F, dynamic changes in mRNA (E) and lncRNA (F) expression in six cryptococcal meningitis patients after antifungal treatment; ****P < 0.0001, (G) the change of LAT titer after treatment; (H) ROC analysis for evaluating the diagnostic value of DPY19L1p1 for cryptococcal meningitis. CN, cryptococcal meningitis; HC, healthy controls

Nine mRNAs and seven lncRNAs were randomly selected for the real‐time PCR validation of relative expression in PBMCs from twenty patients with cryptococcal meningitis. The mRNAs (Figure 5A,C) for cathelicidin antimicrobial peptide (CAMP), lactoferrin (LTF), OLR1, bactericidal/permeability‐increasing protein (BPI), cathepsin G (CTSG), peptidoglycan recognition protein 1 (PGLYRP1), arginase 1 (ARG1), olfactomedin 4 (OLFM4), and carcinoembryonic antigen‐related cell adhesion molecule 8 (CEACAM8,also known as CD66b) were found to be more highly expressed in cryptococcal meningitis patients than in healthy controls. The expression of the lncRNAs (Figure 5B,D), ECRP, LINC00968, DPY19L1p1, DEFA8P, and DEFT1P2 was higher but the expression of the lncRNAs DDX11L10 and MTMR9LP was lower in cryptococcal meningitis patients than in healthy controls, which was consistent with the chip analysis results. Furthermore, we analyzed the dynamic changes in these lncRNAs and mRNAs in six patients (P2, P6, P9, P10, P11, and P12) before and after effective antifungal treatment (voriconazole 4 mg/kg bid and fluorocytosine 100 mg/kg·per day). DPY19L1p1 showed a significant decrease after treatment (Figure 5E,F), which was consistent with titer changes (Figure 5G). Then, the receiver operating curves (ROC) were drawn for evaluating the diagnostic potential of DPY19L1p1 for cryptococcal meningitis, which revealed that DPY19L1p1 was able to discriminate between patients and healthy controls with an AUC_ROC_ of 0.9389 (Figure 5H); *P* < 0.0001.

### 3.7 Functional prediction of lncRNA DPY19L1p1 acting in a *cis* or *trans *manner

**Figure 6:**
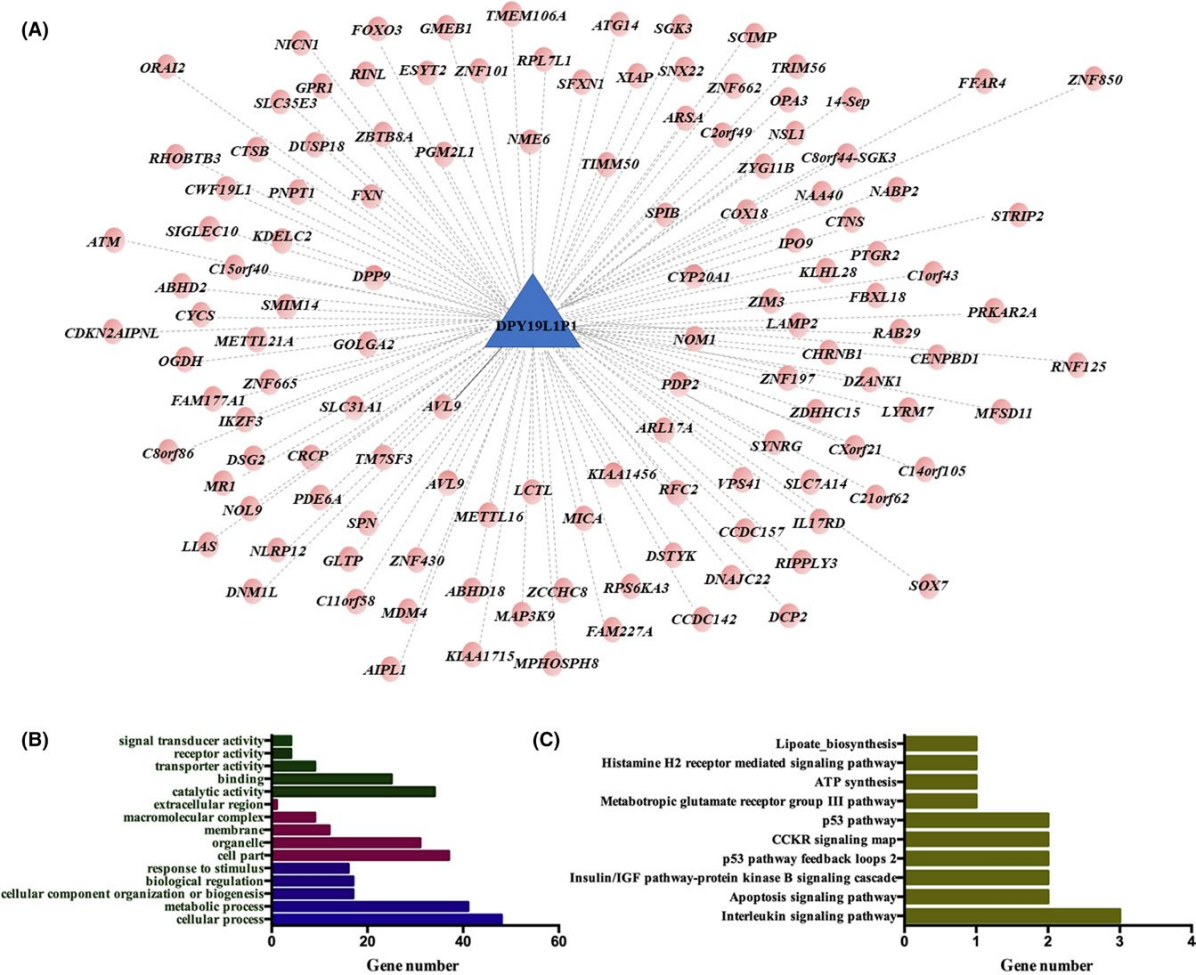
Target prediction for lncRNA DPY19L1p1. A, Targets regulated by *trans* or *cis *mechanisms; B, KEGG analysis of predicted targets; C, GO analysis of predicted targets

LncRNAs regulate genes of interest mainly in a *trans* or a *cis* manner. *Cis*‐regulated genes were selected within a 10 kb distance. LncRNA targets are shown in Figure 6A. One *cis* target gene, namely, AVL9, and one hundred and twenty‐four *trans* target genes were predicted. The top five pathways (Figure 6B,C). involving these target genes were as follows: interleukin signaling pathway, apoptosis signaling pathway, insulin/insulin growth factor (IGF) pathway‐protein kinase B signaling pathway cascade, p53 pathway feedback loops, and cholecystokinin receptor (CCKR) signaling map.

## 4 DISCUSSION

Cryptococcal meningitis is a severe, difficult‐to‐cure disseminated fungal disease. Although numerous studies showed the interplay between human hosts and *C. neoformans, *the overall alteration of gene expression in cryptococcal meningitis patients is unknown. To our knowledge, this study is the first to identify the systemic aberrant expression of lncRNAs and mRNAs in patients with cryptococcal meningitis; thus, our results can provide a broader understanding of the interplay between the host and *Cryptococcus*. In the present study, 201 upregulated and 124 downregulated mRNAs and 263 upregulated and 234 downregulated lncRNAs were identified based on a cutoff value of a twofold change in expression and a *P*‐value of <0.05. The main pathways in which these differential mRNAs were involved were asthma, one carbon pool by folate, allograft rejection, biosynthesis of unsaturated fatty acids, and p53 signaling pathway. Most of the lncRNA differentially expressed in cryptococcal meningitis patients was intergenic lncRNA. A total of 305 coexpression relationships were found between 108 lncRNAs and 87 mRNAs. LncRNA DPY19L1p1 was found to be highly expressed in cryptococcal meningitis patients via PCR validation and tended to decrease after antifungal treatment. In addition, ROC analysis showed DPY19L1p1 had an AUC_ROC_ of 0.9389, indicating an excellent diagnosis potential. Furthermore, the target genes of DPY19L1p1 in *cis* or *trans* regulation were predicted, and most were involved in immune‐related pathways such as the interleukin signaling pathway.

LncRNAs, a large class of noncoding RNAs, are known to be key regulators in many cellular activities, including chromatin remodeling, transcription, splicing, mRNA stabilization, protein translation, and protein translocation.^14^ Several lncRNAs regulate immunogene expression in response to pathogens, and their expression level is dynamically regulated by the interaction between host and microbes.^15^ Aberrant host lncRNA expression was observed upon viral, bacterial, and fungal infection in vitro.^16^, ^17^ Furthermore, some pathogens may utilize host‐expressed lncRNAs to decrease the host immune response.^19^ For the first time, we described the aberrant expression of lncRNAs in cryptococcal meningitis patients; however, whether the aberrant expression was the cause or effect of the cryptococcal infection was unclear. Most of the lncRNAs and expression changes reported herein, such as the upregulation of ECRP, DPY19L1p1, DEFA8P, and DEFT1P2 and the downregulation of DDX11L10 and MTMR9LP, are poorly studied to date, although LINC00968 has been previously reported to be involved in oncogenesis by activating the PI3K/AKT/mTOR and Wnt signaling pathways in vitro.^20^, ^21^ Therefore, the exact involved mechanism of these lncRNAs needs further research.

As lncRNAs with similar functions can interact or present similar network data profiles,^22^, ^23^ a coexpression network between lncRNAs and mRNAs was constructed to predict the potential function of lncRNAs. A total of 305 coexpression relationships were found between 108 lncRNAs and 87 mRNAs. Many key immune regulators, such as SMAD6, were connected to several lncRNAs. During microbial defense, SMAD proteins are activated to induce a protective inflammatory response and are essential for immune system balance.^24^ Another method to predict the function of lncRNAs is lncRNA classification^25^ by type, including intergenic, exonic sense, exonic antisense, intronic sense, intronic antisense, and bidirectional, based on the genomic location. In this study, intergenic lncRNA comprised the majority (nearly 50%) of both the upregulated and downregulated lncRNAs. Although other classifications of lncRNAs remain unclear to date, intergenic lncRNAs have been shown to regulate gene levels via both transcription‐dependent and transcription‐independent mechanisms.^26^ Therefore, as intergenic lncRNAs accounted for the majority of differentially expressed lncRNAs in patients, lncRNAs possibly play a regulatory role in cryptococcal meningitis.

In addition to protein‐noncoding RNAs, most of the protein‐coding genes identified by PCR, including CAMP, LT, CTSG, OLR1, BPI, PGLYRP1, CEACAM8, and OLFM4, which were highly expressed in this study, were found for the first time to be involved in cryptococcal meningitis, although these genes were already known to be involved in antimicrobial and inflammatory responses.^27^, ^28^ Notably, most of the overexpressed genes in our study were related to the NF‐kappaB pathway, consistent with a previous report that the NF‐kappaB signaling pathway can be manipulated by *C. neoformans *within macrophages.^39^

The effective defense against microbes depends on the elaborate collaborative function of the innate and adaptive immune systems. To this end, cytokine signaling could shape the outcome of cryptococcal infection;^40^ the abundant production of Th1‐, Th17‐, and M1 (classical activation)‐related cytokines (such as IFN‐gamma and TNF‐alpha) protect the host, while a shift to Th2‐ and M2 (alternative activation)‐related cytokines (such as IL‐4 and Arg1) was related to increased susceptibility to cryptococcal infection. Our study showed that ARG1 was noticeably overexpressed in patients compared to healthy controls, consistent with the results of previous studies showing M2 activation in cryptococcal meningitis patients.^41^ Although the asthma and p53 signaling pathways were previously reported to be related to cryptococcal infection,^39^, ^42^ new related pathways, including the one carbon pool by folate, allograft rejection, and biosynthesis of unsaturated fatty acids pathways, were identified in this study. These novel pathways furnished another perspective on the immune response triggered by cryptococcal infection.

The dynamic changes in these differentially expressed mRNAs and lncRNAs were also explored. Interestingly, the expression of DPY19L1p1 significantly decreased after antifungal treatment. In addition, ROC analysis showed DPY19L1p1 had an AUC of 0.9389, which indicated that DPY19L1p1 may not only be an indicator for treatment evaluation but also for disease diagnosis. However, these results still need to be confirmed by a larger sample size. Because lncRNAs are important*trans* and *cis* regulators, *trans* and *cis* targets of DPY19L1p1 were predicted to suggest the potential involved pathways. One *cis* target gene and one hundred twenty‐four *trans* target gene were predicted, and their related pathways were explored.^43^ Most target genes of DPY19L1p1 were involved in immune‐related pathways such as the interleukin signaling pathway and p53 pathway feedback loops, indicating that DPY19L1p1 may be involved in the host antimicrobial response against cryptococcal by targeting these immune‐related pathways. However, functional experiments are needed to validate this hypothesis.

## 5 CONCLUSION

In conclusion, for the first time, the aberrant expression of lncRNAs in the patients of cryptococcal meningitis was described, and new involved pathways were identified. Moreover, the results indicated that lncRNA DPY19L1p1 could be used not only in treatment evaluation but also for disease diagnosis. Our study provides new perspectives of the host immune response in cryptococcal meningitis and may aid in future immune‐based therapy research.

## CONFLICT OF INTEREST

The authors declare there are no conflicts of interest.
